# A Disability Bioethics Reading of the FDA and EMA Evaluations on the Marketing Authorisation of Growth Hormone for Idiopathic Short Stature Children

**DOI:** 10.1007/s10728-020-00390-1

**Published:** 2020-02-13

**Authors:** Maria Cristina Murano

**Affiliations:** Linköping University, Campus Valla Hus Key, 58183 Linköping, Sweden

**Keywords:** Short stature, Growth hormone treatment, Children, Disability bioethics, European Medicines Agency, Food and Drug Administration

## Abstract

The diagnosis of idiopathic short stature (ISS) refers to children who are considerably shorter than average without any identified medical reason. The US Food and Drug Administration (FDA) authorised marketing of recombinant human growth hormone (hGH) for ISS in 2003, while the European Medicines Agency (EMA) refused it in 2007. This paper examines the arguments for these decisions as detailed in selected FDA and EMA documents. It combines argumentative analysis with an approach to policy analysis called ‘What’s the problem represented to be’. It argues that the FDA presents its approval as an argument for equity of access to the treatment (given that hGH was already authorised for other indications), describing short stature as a potential disadvantage, and assuming that height normalisation is a clinically meaningful result. The EMA, instead, refuses marketing authorisation with an argument that there is an imbalance of risks and benefits, describing ISS as a healthy condition, and arguing that hGH should provide some psychosocial and/or quality of life benefits to children with ISS other than height gain. This paper then discusses how these arguments could be read through different models of disability, particularly through the medical model of disability and the relational, experiential, and cultural understandings of disability.

## Brief History of Growth Hormone Treatment

Growth hormone treatment (hGH) has been used since the late 1950s to treat children with growth hormone deficiency (GHD), which is commonly described as a medical condition due to the impaired secretion of growth hormone. At first, growth hormone was extracted from the pituitary glands of cadavers, a technique proved to be dangerous three decades later: at least 226 children around the world contracted Creutzfeldt–Jakob syndrome, an incurable neurodegenerative disease, as a result of using this technique [5]. The introduction of recombinant hGH in 1985 allowed increased safety, greater supply, and expanded access. Over time, hGH has been approved for paediatric conditions other than GHD that are known to be associated with short stature, including Turner syndrome, small for gestational age, chronic renal insufficiency, and Prader–Willi syndrome.

One indication raised a unique controversy: idiopathic short stature (ISS). This defines children who are shorter than average, and whose predicted adult height is below 1% of the height of all adults of their sex and population group. While in 2003 the Food and Drug Administration (FDA) approved the use of hGH to treat children with ISS, in 2007 the European Medicines Agency (EMA) refused its use for this indication. Recently, the Pediatric Endocrine Society (PES) recommended a conservative approach to prescribing hGH for ISS in the US [20, 22]. The PES defines ISS as a “controversial indication” for hGH [22, p. 362] because, besides limits in diagnostic accuracy (e.g. some children might have a subtle form of GHD), long-term safety is uncertain (i.e. the possible increased risk of cancer, cerebrovascular disease, and metabolic side effects have not been sufficiently documented), and “the degree” of disability of children with ISS is “not clear” [22, p. 386].

This paper has two aims. First, it identifies and examines the arguments for and against the marketing authorisation of hGH for the indication of ISS appearing in the selected documents of the FDA and the EMA, focusing on explicit and implicit assumptions and presuppositions concerning the understanding of short stature and the use of hGH treatment for the indication of ISS. Second, it discusses how these arguments could be read through different models of disability, particularly through the medical model of disability and the relational, experiential, and cultural understandings of disability, and how these readings help shed further light on assumptions within these documents. The analysis combines argumentative analysis [24, 39] with an approach to policy analysis called ‘What’s the problem represented to be’ (WPR) [2, 4] and draws on critical disability studies.

## The US and EU Drug Regulatory Agencies

This paper focuses on selected FDA and EMA documents that assess the efficacy and safety of hGH treatment for the paediatric indication of ISS. While at the time of these evaluations, hGH was already marketed both in the US and in the EU to treat other indications (such as GHD and Turner syndrome), the documents analysed here focus on the decision for or against the marketing authorisation of hGH for the paediatric indication of ISS.

### The Food and Drug Administration

As an agency of the US Department of Health and Human Services, the FDA is part of the executive branch of government. As such, it cannot make new laws but can issue regulations designed to implement laws passed by Congress. Founded in 1906 (and named the FDA in 1930), the FDA is responsible for protecting and promoting public health through the control and supervision of food, drugs, and other products, such as tobacco and nutritional supplements. The FDA evaluates new drug applications (NDA) and must approve a drug for a specific indication—and consider its safety and the efficacy—before the drug can be marketed. After NDA approval, the sponsor must review and report every known adverse event to the FDA [15].

### The European Medicines Agency

The EMA is a scientific agency founded in 1995 to protect and promote public and animal health through the scientific evaluation of medicines for human and veterinary use. In the EU, the vast majority of innovative medicines must follow a centrally authorised procedure coordinated by the EMA (for example, drugs containing a new active substance to treat specific conditions, such as HIV and cancer, drugs derived from biotechnology processes, or drugs for rare diseases). Under the centralised authorisation procedure, the EMA offers *centralised* Marketing Authorisation (MA) in the sense that drug companies make a single application in order to validate MA throughout the EU, the European Economic Area (EEA), the European Free Trade Association (EFTA), Iceland, Norway, and Liechtenstein. However, the EMA provides *decentralised* scientific assessment because it relies on a network of 4500 experts throughout the EU. The EMA carries out a scientific assessment of the application made by the pharmaceutical companies and recommends whether the drug should be marketed or not. The European Commission uses the EMA evaluation to render a binding decision on marketing the product in the EU [13].

### The Agencies’ Assessments of hGH Treatment for the Indication of ISS

*Eli Lilly and Company* in 2003 and *Ipsen Ltd.* in 2006 made their request respectively to the FDA and EMA to market their own hGH brands, *HUMATROPE* and *NutropinAQ*, for the indication of ISS. These products contain the same pharmaceutical compound (somatropin). Both companies named the indication as “non-growth hormone deficient short stature” (NGHDSS). The FDA document uses this label, while the EMA uses “severe ISS”. Both labels indicate the same paediatric condition, the medical indication of ISS, namely height that is considerably shorter than average without any identified medical cause. This diagnosis can only be made by excluding any known pathology related to short stature. However, each industry application proposes specific measurement criteria, and both sets of criteria are more restrictive than the definition of ISS, which includes all children below − 2 SD from average height-for-age on a standard height curve.^1^ This paper consistently refers to such a condition as “ISS”, the acronym most commonly used in the literature on this subject [6, 14], in line with the clinical usage [9], and the one that is preferred in the more recent PES recommendations on hGH [20, 22].

## Methods

To collect the agencies’ documents concerning the assessment of hGH for the indication of ISS, I used different strategies according to the agencies’ decision-making processes and public information policies. In both cases, I first searched their websites for ‘somatropin’, the international non-proprietary name for the drug (i.e. the official and generic name for a recombinant form of hGH).

The FDA approved the use of *HUMATROPE* for children with ISS during the Endocrinologic and Metabolic Drugs Advisory Committee meeting on June 10, 2003. On the FDA website, I downloaded material available on this meeting: the notice of the meeting, the draft agenda, the draft questions, the committee roster, the consultants-guests roster, briefing information, the PowerPoint presentations made during the meeting (both by the pharmaceutical company and the scientific advisory group), a brief video of a child’s testimony, the index, and a transcript. The EMA website contains limited public information on the discussions that led to the final decision [10], because the EMA followed the *decentralised* decision-making process through several in-person and online discussions among European experts. I therefore made an online request for copies of the rapporteurs’ assessment reports, the minutes of the Scientific Advisory Group (SAG)—composed by European experts—and the company’s request for extension. These 13 documents were released in several batches (from January until June 2016).

After reviewing the collected documents, I selected those that contained considerations on the relevance of short stature and the reasons why hGH treatment should be approved or refused, besides technical evaluations of safety and efficacy. I then analysed the FDA’s briefing document [16], and the EMA’s SAG meeting minutes [9], the opinion on the marketing authorisation [11], the public document with questions and answers on the recommendation for refusal of change in marketing authorisation [10], and the withdrawal assessment report [12].

According to the material collected and the aims of this article, I combined argumentative analysis [24, 39] and an approach to policy analysis called ‘What’s the problem represented to be’ (WPR) [2, 4]. While I aim to reconstruct the argumentations provided as clearly as possible [24], I do not aim to uncover fallacies (understood as mistakes in the argument) in a strictly logical sense, but to explore and critically discuss the norms, values and assumptions of these arguments. WPR is a post-structural approach to analysis that understands policies not as ways to solve problems, but as ways to represent and shape them in specific ways. Therefore, WPR aims to “dig deeper than usual into the meaning of policies and into the meaning-making that is part of policy formulation” [2, p. vi]. On the one hand, WPR aims to *identify* and *reconstruct* the ways the documents represent (or problematise) the object of discussion; on the other hand, it *interrogates* them [4, p. 19]. The concept of problematisation is used in two ways in WPR. First, it refers to the “the ways in which particular issues are conceived as ‘problems’, identifying the thinking behind particular forms of rule” [2, p. 30]; second, problematising means “to interrogate” [2, p. 30].

WPR considers policy as a “cultural product” in the anthropological sense that decision-making “takes shape within specific historical and national or international contexts” [2, p. ix]. It is a flexible analytical method that stimulates *critical* thinking and *self*-*reflectivity* and that can be applied to texts that are not formally recognised as policy, but that belong “to the full range of articulations of ‘what to do’ by policy-making institutions” [28, p. 64]. WPR encourages a *critical* approach as it aims to analyse how the documents represent (make sense of) the problem at stake (i.e. whether hGH should be marketed for ISS or not), and focuses on implicit and explicit assumptions and presuppositions underlying the representation of the problem. It is a *(self)reflective* approach because it entails interrogating the documents with a list of questions^2^ and then probing one’s own representation of the problem with the same questions. WPR encourages reflection on the broader implications that policies’ formulations might have on society,^3^ and this manuscript focuses on the ethical implications.

In what follows, I was inspired by the WPR forms of questioning to first reconstruct separately and as clearly as possible the arguments that arise from the FDA and the EMA documents, while focusing on explicit and implicit assumptions and presuppositions on short stature and the justification of hGH. I then interrogated these arguments by drawing upon critical disability studies.

## The FDA’s Argument of Equity

The FDA recommends the approval of marketing authorisation of *HUMATROPE* for the paediatric indication of ISS, advancing an argument based on the idea of equity. Given that hGH has been approved for some non-GHD paediatric indications to “treat growth impairment and not the underlying condition” [16, p. 24], and that hGH has similar safety and efficacy profiles for these conditions and ISS, hGH should be approved for the indication of ISS. The FDA describes short stature as a disadvantage [16, p. 27] and the approval of hGH for this indication as a way to “correct the current inequity in treatment availability” [16, p. 21]. In other words, the FDA argues that given that hGH is currently approved for some children without GHD in order to address the disadvantage of short stature, it would be unfair to exclude those with ISS from treatment. This argument is built on two main assumptions and presuppositions: a) short stature is a potential disadvantage, regardless of its medical explanation, b) hGH promotes equity by preventing discrimination based on height.

### The Potential Disadvantage of Short Stature

The FDA briefing document highlights a recurring analogy between the indication of ISS and the other four non-GHD conditions that at that time were already approved for the generic drug, somatropin. These conditions are chronic renal insufficiency, Turner syndrome, Prader–Willi syndrome, and children born small for gestational age. Of these, only Turner syndrome was approved for the brand *HUMATROPE*. The FDA document states that children with ISS are shorter than average, have adequate secretion of GH and might have an “equivalent degree of short stature” [16, p. 16] and “the same severity of growth failure” [16, p. 28] as the other non-GHD conditions already treated with hGH. If untreated, children with ISS commonly “achieve adult heights that fall below the adult heights predicted for them during childhood” [16, p. 28]. The analogy between different indications is also suggested by the FDA’s decision to refer to children with ISS using the label proposed by the industry, i.e. “NGHDSS”, sometimes spelled “non-GHD short stature” [16, p. 146], as if ISS would fit under the same category of the other four non-GHD conditions. The FDA document shifts the focus from different aetiologies of short stature to short stature itself, which is described as potentially bringing “disadvantages”:

Children and adults with short stature, irrespective of cause, may have a number of disadvantages in life relative to their normal-stature peers. Short children may be subject to juvenilization, teasing, bullying, exclusion from activities and peer groups and impairment of the normal progression toward independence [32, 40]. In adulthood there may be problems of social isolation, reduced marriage rates, perceptions of lower competence, and ineligibility for certain occupations that have specific minimum height requirements. There are a number of potential employers, such as construction companies, the aviation and aerospace industries, and the military, that have minimum height standards for employees. Furthermore, aspects of daily living such as driving a car, accessing cupboards, using kitchen or bathroom benches or sinks, and using standard height furniture in workplaces may provide additional challenges [16, pp. 27–28].

In other words, the FDA document argues that the potential disadvantages of short stature are more relevant for the assessment of hGH than the cause of short stature. The disadvantages of short stature are, according to the FDA, related to social and professional discrimination, or to limitations connected to objects and/or infrastructures built according to average height.

### HGH Provides Fair Opportunities

The FDA document argues that hGH should be used in children with ISS for the same reason it is used for children with other non-GHD conditions, namely to increase height. While the latter might “have additional problems beyond their growth disturbance (such as ovarian failure in Turner syndrome)”, these problems “are not addressed by somatropin” [16, p. 29]. If hGH is accepted to increase height in some conditions, the document states, in the case of ISS, the “absence of a known etiology for the growth failure does not justify exclusion from treatment” [16, p. 27].

According to the FDA, given that short stature is a disadvantage and hGH makes children grow taller, the height increase provided by hGH is a benefit because it addresses such a disadvantage. Furthermore, the FDA states that previous studies do not show evidence that hGH brings psychological improvement or quality of life increase to children with ISS [16]. Yet the FDA defines the height gain of 7 cm compared to predicted adult height as “clinically meaningful” [16, p. 18] for three main reasons. First, it provides a “similar” height gain as that for Turner syndrome; second, it brings the mean height of children into “the *normal range*” during the treatment; and third, in previous studies, treated patients reached “*normal range*” in adulthood, while those in the placebo group did not [16, p. 18, *emphasis added*].

In this view, if short stature brings disadvantages, the *normalisation* of height is a benefit as it addresses such disadvantages. Therefore, as a matter of equity, children with ISS should be able to opt for a normalized height in the same way as other non-GHD indications. As the FDA states:

Patients with non-GHD short stature are just as short, and just as deserving of treatment as children with GHD, chronic renal insufficiency, Turner syndrome, Prader-Willi syndrome, or children born small for gestational age, and the lack of approved treatment for these children represents a major inequity and unmet need [16, p. 146].

## The EMA’s Argument of an Unfavourable Risk–Benefits Ratio

The EMA recommends refusal of marketing authorisation for the hGH brand *NutropinAQ* for the indication of ISS because its benefits do not outweigh the possible risks. On the one hand, the EMA describes children with ISS as healthy. On the other hand, it states that the height gain provided by hGH is “only modest” [10, p. 2], and stresses both poor knowledge about long-term safety and lack of evidence on the improvements hGH can offer children with ISS in terms of quality of life or psychological well-being. In my reading, this argument, as it is presented in the selected documents, is based on two main assumptions: (1) the distinction between different kinds of short stature, (2) the understanding of hGH as an unnecessary cosmetic intervention.

### Different Kinds of Short Stature

The Scientific Advisory Group meeting minutes state that the EMA members draw a distinction between three different kinds of short stature: (1) ISS and other non-GHD conditions, (2) children who are and those who are not referred to endocrinologists because of their height, (3) short stature and “extreme short stature” [9, p. 3]. First, although short-term safety of hGH for ISS is similar to that for GHD and Turner syndrome, the assessment of hGH should not be based “on analogies,” but evidence is needed for each indication [9, p. 6]. The category of ISS is defined as indicating children who “are *normal* in terms of health, but *abnormal* in terms of height” [*emphasis added*, 9, p. 2]. The document states that defining ISS as a healthy condition requires a different set of considerations than those made for “well-defined disease states” [9, p. 3], such as GHD and Turner syndrome. Second, children who are referred to endocrinologists because of their height might be more likely to experience psychosocial problems than those not referred [12]. Third, even if “extreme short stature could be considered a form of handicap” [9, p. 3], there is no evidence that being short affects the psychological well-being of children with ISS, nor that hGH might improve it [9].

The document indicates that during the meeting the experts highlighted that terminological choice plays a role in the evaluation of the acceptability of the treatment. While the industry *Ipsen Ltd.* uses the term NGHDSS, the EMA opts for “severe ISS” because ISS conforms to the “current clinical usage” [9, p. 4]. Moreover, according to the document, referring to ISS clearly rules out other non-GHD conditions [9]. However, the document refers to the concern of some EMA experts that ISS might be understood as a “disease label” [9, p. 2] implying a recommendation for treatment. To avoid this misunderstanding, they would have preferred the label “short healthy children” [9, p. 2], because it clarified, for them, that children with ISS represent a “marked statistical deviation from the population mean,” but do not necessarily require treatment [9, p. 2]. Though some members proposed the label “normal short stature” [9, p. 2], it was rejected for its implication that no child with ISS should be treated.

### Given Current Knowledge, hGH is an Unjustifiable Cosmetic Intervention

In the document available online, the EMA states that, given the current state of knowledge, the benefits provided by hGH treatment for children with ISS do not outweigh the possible risks for three main reasons. First, the final height gain of 6–7 cm is “only a modest benefit” [10, p. 2]. Some members even question whether children would gain 6-7 cm in adulthood because the study presenting this result did not have a control group [9]. Second, there is no proof that the treatment improves the psychological and social well-being of children with ISS [10]. And third, there are concerns that long-term side effects might occur, such as tumours and diabetes [10]. The meeting minutes state that, because of the current limitations in defining the circumstances in which children with ISS might benefit from hGH treatment, more evidence is needed before approval can be granted [9].

The meeting minutes therefore highlight two points. First, hGH raises a fundamental concern about the aim of the treatment, as it would assign “healthy” children to long-term medical therapy for “cosmetic reasons” [9, p. 2]. This statement emphasises that EMA experts disagree with the rationale of treating “healthy” children with the sole aim of making them grow taller. Second, hGH treatment is not “acceptable” for children with ISS when we consider its limited benefits and possible side effects [9, p. 2]. This second point leaves open the question of what argument the EMA would offer if there were no proven negative consequences or if the height increase were to prove greater for children with ISS.

## Putting the Arguments into Context

To recap, while the final *arguments* of the FDA and the EMA are one of equity and one of an imbalance of risks and benefits, in my reading, the *problematisation* of the FDA is that since hGH treatment aims at increasing height, it should be made available to children with ISS. This problematisation is based on two main assumptions: (1) short stature is potentially a disadvantage for children with ISS in the same way as it might be for other non-GHD conditions, (2) approving hGH to normalise height is a way to provide fair opportunities. The EMA, on the other hand, would approve hGH if it provided some psychosocial benefits besides height gain. This problematisation is based on the assumptions that: (1) children with ISS are short but healthy, (2) hGH is a cosmetic intervention for ISS that does not have sufficient evidence of benefit.

It should be noted that the final decision was taken through different decision-making processes, and the discussions presented some internal disagreements. The FDA took its decision during the plenary meeting, in which also the pharmaceutical company gave a PowerPoint presentation of its studies’ results and at least one brief video featuring a treated child (who was enthusiastic about hGH). However, although eight out of ten FDA experts were in favour of marketing authorisation, only three of the ten voting members of the committee were convinced that the drug was “clinically meaningful” in the sense that the height gain provided would benefit treated children [23]. The EMA, instead, took its decision after a series of meetings and online discussions among experts across Europe. During the first meeting there was a general consensus on approving hGH for ISS, but concerns prevailed in the following discussions [9]. Eventually, 14 out of 24 experts voted against the marketing authorisation of hGH for the indication of ISS [11]. The remaining ten experts would have approved it because, according to them, height gain was well documented and there was no evidence of long-term side effects from available data on treated children [11].

More recently, in the US, the PES has evaluated the quality of evidence of hGH for the indication of ISS, applying healthcare quality improvement strategies, which emphasise the need for evidence-based decision-making [20]. The PES guidelines recommend “against the routine use of GH in every child” with ISS [22, p. 362]. They propose a form of problematisation similar to that of the EMA, claiming that children with “ISS are healthy and will continue to be healthy without treatment” [22, p. 386] and that hGH should provide not only height gain but also psychological well-being. Among other recommendations, they propose an approach for determining the need for hGH for each individual child:

Potentially, the degree of physical and/or psychosocial disability that an individual child suffers due to short stature could be used to determine which children should receive GH therapy [1, 22, p. 387].

In the next section, I discuss how the FDA and EMA arguments could be read through critical disability studies and how different understandings of disability can provide alternative ways of problematising both the understanding of short stature and possible ethical justifications of hGH for children with ISS.

## Disability Bioethics Considerations

While both agencies acknowledge an ethical dimension to their decisions [16, p. 30; 9, p. 6], their arguments can be read as having different ethical implications, depending on how disability is understood. The PES guidelines state that after the FDA marketing authorisation, many children not fitting the definition of ISS (or any other approved drug indications) have sought medical treatment [22]. This suggests that many people might have interpreted the FDA approval as implying that all kinds of short stature potentially lead to disadvantage. The EMA decision, instead, might be interpreted as indicating a sharp distinction between the justification for hGH for children with ISS and that for other disease conditions. Previous research, however, has argued that the distinction between pathological and healthy short stature might be arbitrary [30]. In what follows, I discuss how the agencies’ arguments might be read as taking different ethical viewpoints if considered based on the medical, relational, experiential, and cultural understandings of disability.

### The Medical Understanding of Short Stature

The FDA refers to shortness in medical terms such as “growth impairment” [16, p. 24], “growth disturbance” or “growth failure” [16, p. 29]. At the same time, it describes short stature as a potential psychosocial disadvantage due to social discrimination and the current functioning of society. One way to understand the FDA approach can be through the medical (or individual) model of disability. The medical model understands disability in medical terms, as a functional impairment or limitation, and as a problem inherent to the individual [34]. Oliver and Barnes [31] state that, even if the medical model describes the reason for the disadvantage as social, “impairment is seen as the underlying cause of ‘disability’ or ‘handicap’” [31, p. 19]. The individual model is, according to Oliver and Barnes [31], strictly tied to medical intervention: if the problem is described as being in the individual, the solution is to intervene with medicine to prevent, treat, or cure the problem [31]. Similarly, the FDA argument can be read as implying that the potential sociocultural problem of short stature is due to height. It is because of height that short children might encounter social discrimination and disadvantage. Therefore, the use of hGH to normalise height is understood as a way of providing fair opportunities. The way in which the medical problem conceptualises the problem at stake (in the FDA case, short stature) makes treatment (i.e. hGH) appear ethically justified (i.e. a matter of fairness).

The medical model is also called the individual model because it describes the problem as intrinsic to the individual [31, 34]. Even if the EMA describes the indication of ISS as healthy, and refuses the treatment for this indication because of the lack of evidence of sufficient benefits, it might be read as describing short stature as a problem to be investigated at the individual level. It states, for example, that it approves hGH for other non-GHD conditions, and differentiates children with ISS from those referred to endocrinologists because of their short height, who are more likely to experience psychosocial problems [12] and from those who have “extreme short stature” who may be seen as handicapped [9, p. 3]. The EMA also states:

Whilst accepting the clinical view that individual children with ISS might in special circumstances benefit from hormone therapy, it was noted that such circumstances were almost impossible to define adequately [9, p. 6].

The EMA problematisation, understood through the medical (individual) model goes like this: hGH might be justified for individual children who might benefit from it, but because of limitations in evidence of benefit for the indication of ISS, hGH should not be approved for this indication. While the EMA states that it is difficult to define in which circumstances children with ISS would benefit from hGH, its argument can be read as if short stature can become troubling to the extent that it qualifies as a special circumstance, and this qualification, in turn, would shift the risks-benefits balance. Under such special circumstances, the height increase provided by hGH would become justified.

If the FDA and the EMA forms of problematisation are read through the medical model of disability, the implication seems to be that, under certain circumstances, children with short stature might *benefit* from hGH. In other words, the agencies would assume a similar epistemological approach: it is not merely possible to treat short stature, but *there is* or *might be* a kind of short stature that might *benefit* from treatment. While the FDA argues that this is the case for the indication of ISS, the EMA argues that this might be the case only under special circumstances. In both cases, hGH is justified if children might benefit from a height increase.

The medical model, however, has been criticised by many and is seen as opposed to the social model of disability. According to the social model, disability is the result of social disabling barriers: it is the limited access to social and cultural life, due to social discrimination, stigma, and practical obstacles, that defines the disability [31]. Following this model, short stature should be understood not as an intrinsic problem of the individual child (either in terms of height measurement or psychological well-being), but as a problem contingent on social discriminations and/or disadvantage. For example, if cars are built according to average height, smaller cars should be available for short people. Similarly, employers should adapt the working environment to make it accessible to short people. In this view, therefore, the epistemological approach is that short stature is not a problem of the individual. Therefore, hGH is not justified for children with ISS.

### The Relational, Experiential, and Cultural Understandings of Short Stature

Although the social model has been powerful in promoting political and social change and disability rights, it has been criticised by scholars who note that it fails to acknowledge aspects such as the experiential, relational, and cultural dimensions of disability [31, 36], as well as the health dimension [33]. As an alternative, Fougeyrollas and Beauregard [17] suggest an ‘interactive/relational process’ approach and, following the World Health Organization International Classification of Functioning, Disability and Health [37] propose an understanding of disability as “an interaction between personal characteristics and environmental factors” [17]. They do not see disability as a fixed state or individual characteristic but as a process influenced by multiple external and internal factors. This model takes as its starting point a human development model applicable to everyone and stresses the fact that development happens over time, through a dynamic and complex process. This model does not offer a clear answer as regards the justification of treatment, but it provides detailed “definitions, taxonomies and measurement scales for life habits, environmental factors and personal factors conceptual domains”, and it might help to identify what kind of problem(s) the child might have [26].

Also the experiential and cultural understandings of disability define disability as a complex, dynamic, and multidimensional experience [19, 29, 41]. Moreover, these accounts stress the fact that there might be very different kinds of disability, different ways in which people might experience the same disability, and one’s experience might even change gradually over time. Disability is not necessarily an abrupt and disruptive event, but in some cases, like cerebral palsy, it might be a gradual experience [29]. The way one experiences one’s own condition is not merely determined by the condition in itself (for example, lacking parts of the face, such as the nose or ears), but, looking at one’s embodied interaction with others and the world, it might vary with changes in situations, relationships, and one’s emotions [41, 42]. Also in this case, there is no clear suggestion for a justification of medical intervention, but for an alternative way of seeing. For instance, an empirical study attentive to the embodied experience shows that, contrary to what some might assume, women after mastectomy might not welcome breast prostheses and they might prefer not to undergo reconstructive surgery [35].

Garland-Thompson [19], taking a cultural approach, suggests that disability can be understood as not denoting a specific category of people, because each of us “evolve[s] into disability. Our bodies need care and assistance to live. Disability is the essential characteristics of being human” [19, p. 328]. According to Garland-Thomson [18], understanding disability within a continuum of human experience reveals the many contributions that disability brings to the world, and shows that medical intervention to change it or eliminate it might not be justified [18].

The relational, experiential, and cultural accounts all undermine the causal link between disability and the idea of a specific understanding of a problem because they take into account the complexity and dynamics of the various factors that might co-shape human lives. Understanding short stature by using these models would not provide any clear answer on the justification of hGH, but would encourage examination of short stature within the complex, dynamic and multidimensional process of human growth. These accounts problematise the idea that a form of short stature *exists* that *benefits* from treatment. They put forward an understanding of the benefits of hGH in terms of a hypothesis rather than facts. The hypothetical formulation increases awareness of the fact that choosing for or against hGH for a child with ISS implies a certain degree of uncertainty about the effects that this might have on the child’s life,^4^ while reminding us to be attentive to many other aspects that influence the choice.

This manuscript contributes to bioethical discussions as it suggests not only that the assumptions and presuppositions analysed above have shaped the formulation of the FDA and EMA arguments, but also that these arguments might be understood differently and have different ethical implications according to one’s assumptions and presuppositions. They could be read, I argue, through the inputs of the relational, experiential, and cultural understandings of disability. The fact that the FDA leaves open the possibility of treating the indication of ISS could be read as an invitation to evaluate height as a complex, dynamic and multidimensional experience of the growing child. The EMA, on the other hand, leaves the special circumstances in which height might be seen as a problem open to interpretation. This approach might be read as acknowledging the complexity at stake and therefore could be used to welcome the PES suggestion to evaluate whether to treat the individual child through disability, not in terms of (quantitative) degrees of disability but with an eye to the qualitative dimensions that the experience, the cultural understandings, and the relationships of children with short stature might entail.

## Conclusion

My analysis of the FDA and EMA arguments has focused on the ways the selected documents represent the problem of whether or not to approve the marketing authorisation of hGH for the indication of ISS. I sought to clarify the understandings of the problem conveyed by the documents, focusing on underlying assumptions and presuppositions about short stature and their justifications of hGH treatment for the indication of ISS. I then drew on disability literature to discuss different ways in which the FDA and EMA arguments can be read and the ethical justification of hGH for ISS interpreted. I have argued that the relational, experiential, and cultural models of disability suggest that the existence of certain kinds of short stature that benefit from hGH could be seen not as a fact, but a hypothesis that embraces the uncertainties of using hGH for children with ISS. While the agencies based their decisions on the indication of ISS, the implications of short stature for children might be complex, dynamic, and multidimensional in the same way as human growth. Furthermore, the choice of hGH is not determined merely by issues of efficacy and safety, but requires an examination of the values that we might want to convey with that choice. In particular, we should be careful not to interpret these kinds of decisions on medications in a way that reinforces unjustified discriminatory attitudes more widely across society.

HGH treatment is now technically possible. This technical possibility does not necessarily imply that a category of short children needing treatment exists. It merely means that some people would opt for hGH treatment for several reasons, such as: parental concerns, an aesthetic preference to be average height (on the part of the child or parents), or a desire to fit in by whatever means possible. The dimension of uncertainty, due to an understanding of growth as a process over time, however, should be taken into account in ethical evaluations. Although families and doctors might see short stature as a potential problem at the time of decision-making, they should be reminded that the child (potentially) receiving the treatment is *in becoming*, many possibilities are open, and different problematisations of both the relevance of short stature and the justification of the treatment are possible.

## Footnotes

1 *Eli Lilly and Company* refers to children who are below − 2.25 SD, which “corresponds [in adults] to 5 feet, 2.9 in. (159.8 cm) in males and 4 feet, 10.5 in. (148.5 cm) in females” [16, p. 35]. *Ipsen Ltd.*, instead, refers to children with current height below − 3 SD, or those who are below − 2.25 SD and also have decreased growth velocity (below the 25th percentile), leading to a predicted adult height less than 1 SD below targeted height [8].

2 WPR asks six questions: (1) What’s the problem represented to be? (2) What deep-seated presuppositions or assumptions underlie this representation of the “problem”? (3) How has this representation of the “problem” come about? (4) What is left unproblematic in this problem representation? Can the “problem” be conceptualised differently? (5) What effects (discursive, subjectification, lived) are produced by this representation of the “problem”? and (6) How and where has this representation of the “problem” been produced, disseminated, and defended? How has it been and/or how can it be disrupted and replaced? These questions involve some “overlap and repetitions” and therefore “their listing as separate ‘steps’ serves a heuristic function and ought to be treated accordingly” [4, p. 19]. This means that WPR foresees that the analysis will “draw selectively upon the forms of questioning and analysis just described, so long as a self-problematising ethic is maintained” [2, p. 24].

3 WPR has been used to critically analyse the formulation of problems in policies and documents concerning different subjects, such as substance use treatment for migrants and ethnic minorities [7], the representation of men’s violence on women [25], Roma and education [27] and health policies [3]. To the best of my knowledge, this manuscript is the first to use the WPR method with an explicit focus on bioethics.

4 Some studies, for instance, have shown that parents’ anxieties and concerns about the impact of short stature do not necessarily correspond to what their children experience or think of themselves and sometimes overestimate their problems [21]. Some sociocultural norms and beliefs about short stature, instead, have been proved to be supported by partial evidence. For example, studies showing the preference for tall men have mainly been conducted without considering sexual orientation. At least one study that did so shows that the partner’s height preference might differ between heterosexual and non-heterosexual individuals [38].
